# Cytotoxic Evaluation of Elastomeric Dental Impression Materials on a Permanent Mouse Cell Line and on a Primary Human Gingival Fibroblast Culture

**DOI:** 10.3390/ma2030934

**Published:** 2009-08-14

**Authors:** Federica Boraldi, Chiara Coppi, Sergio Bortolini, Ugo Consolo, Roberta Tiozzo

**Affiliations:** 1 Department of Biomedical Sciences, Section of General Pathology, University of Modena and Reggio Emilia, Via G. Campi 287, 41100 Modena, Italy; E-Mail: federica.boraldi@unimore.it (F.B.); 2 Department of Neurosciences, Section of Dentistry, University of Modena and Reggio Emilia, Via del Pozzo 71, 41100 Modena, Italy; E-Mail: chiaracoppi@yahoo.it (C.C.); 3 Department of Neurosciences, Section of Dentistry, University of Modena and Reggio Emilia, Via del Pozzo 71, 41100 Modena, Italy; E-Mail: sergio.bortolini@unimore.it (S.B.); ugo.consolo@unimore.it (U.C.)

**Keywords:** dental impression materials, citotoxicity test, cell culture

## Abstract

The need for clinically relevant *in vitro* tests of dental materials is widely recognized. Nearly all dental impression materials are introduced into the mouth just after mixing and allowed to set in contact with the oral tissues. Under these conditions, the materials may be toxic to cells or may sensitize the tissues. The aim of the present study is to evaluate the potential cytotoxicity of new preparations of elastomeric dental impression materials: A) four vinylpolysiloxanes: Elite H-D Putty and Elite H-D Light Body (Zhermack, Badia Polesine, Rovigo, Italy); Express Putty and Express Light Body (3M ESPE AG Seefeld, Germany) and B) two polyethers: Impregum Penta and Permadyne Penta L (3M ESPE AG Seefeld, Germany). The cytotoxicity of these impression materials were examined using two different cell lines: Balb/c 3T3 (permanent cell line) and human gingival fibroblasts (primary cell line) and their effects were studied by indirect and direct tests. The direct tests are performed by placing one sample of the impression materials in the centre of the Petri dishes at the time of the seeding of cells. The cell growth was evaluated at the 12th and 24th hours by cell number. The indirect tests were performed by incubating a square of 1 cm diameter impression material in 5 mL of medium at 37 °C for 24 hours (“eluates”). Subconfluent cultures are incubated with “eluates” for 24 hours. The MTT-formazan production is the method used for measuring the cell viability. The results indicate that: a) polyether materials are cytotoxic under both experimental conditions; b) among vinylpolysiloxanes, only Express Light Body (3M ESPE AG Seefeld, Germany) induces clear inhibition of cellular viability of Balb/c 3T3 evaluated by direct and indirect tests and c) the primary cell line is less sensitive to the toxic effect than the permanent cell line.

## 1. Introduction

All dental impression materials should accurately replicate oral tissues in terms of accuracy, dimensional stability, elasticity, tear strength, rigidity, reproduction of detail and biocompatibility [1,2,3]. The assessment of cytotoxicity of these materials is a fundamental step in the evaluation of their biocompatibility. The *in vitro* test methods, designed to evaluate the acute adverse biological effects of medical device materials, are regulated by organizations such as the International Organization for Standardization (ISO 10993) [4,5]. In *in vitro* tests, a cell monolayer is grown to sub confluence and then exposed to materials directly or indirectly. The literature reports many observations on the potential cytotoxicity of various dental materials on different cell lines cultured *in vitro* [6,7,8,9,10,11]. On the contrary, only few *in vitro* studies on the cytotoxic effects of dental impression materials on cells cultured *in vitro* are reported [12,13,14,15,16,17]. Between dental impression materials, four types of elastomers are extensively used: polysulfides, condensation silicones, polyethers and vinylpolysiloxanes [3]. They are generally supplied in two paste forms, base and catalyst, and may be dispensed through an auto-mixing cartridge. Although polyethers present many advantages for clinical use, several disadvantages have been reported, including allergic and toxic reactions, contact dermatitis and gingivitis [18,19,20]. Vinylpolysiloxanes are more commonly used [3]. Studies on their toxicity have been contradictory, as they have been variously classified as toxic [12,13,14,15], less toxic [12] or non-toxic [13,17]. The different grades of toxicity, evaluated *in vitro*, depend on the type of culture (primary cell or permanent cell lines), on the type of *in vitro* test (direct or indirect), and on the manufacturing processes of the materials. Generally, impression materials remain in contact with the oral tissues for a short time, typically a few minutes. The toxicity of the dental impression material is especially important when, during an intervention, a fragment becomes entrapped and remains within the gingival sulcus [23] under the suture, during impression making for implants or a surgical prosthesis. This can also occur in implant dentistry, particularly during second stage implant surgery or during single stage surgery. The retention of these fragments can induce a severe inflammatory reaction [24,25], which can result in implant failure.

The aim of the present study was to determine the cytotoxicity of a new generation of impression dental materials on Balb/c 3T3 cells (a permanent cell line) and human gingival fibroblasts (a finite cell line) cultured *in vitro*. Balb/c 3T3 cells are easy to maintain, grow quickly, show good reproducibility and they are simple replicating systems without the specific metabolic potential that the target cells have *in vivo*. Moreover continuous lines are sensitive and useful to test and classify the toxic effect of different materials [26]. Human gingival fibroblasts cultured are characterized by high degree of differentiation and even if they are less homogenous and sensitive than permanent cell lines, they are more comparable in their reaction pattern to oral cavity. Human gingival fibroblasts retain specialized features and can represent a good simulation of *in vivo* conditions, particularly in the case of impression material retention [27].

## 2. Results and Discussion

### 2.1. Direct effect of vinylpolysiloxane and polyether on Balb/c 3T3 proliferation

**Figure 1:**
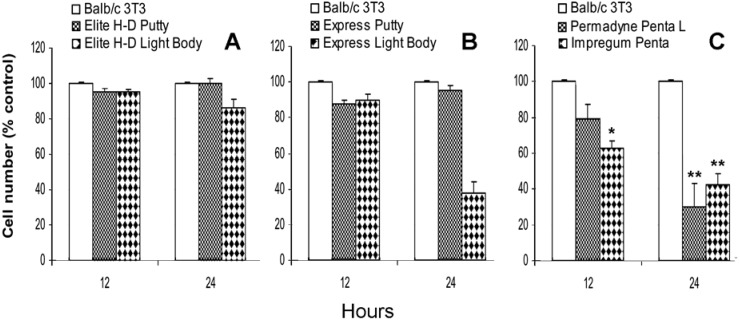
Direct effect of Elite H-D Putty, Elite H-D Light Body (A), Express Putty, Express Light Body (B), and Permadyne Penta L and Impregum Penta (C) on the *in vitro* proliferation of Balb/c 3T3 cells Data are expressed as a percentage of the untreated cells. Each graph averages the results from at least four measurements. *P <0.05, **P <0.01. One way ANOVA test and Bonferroni multiple comparison test.

Figure 1A shows the direct effect of vinylpolysiloxane impression materials: Elite H-D Putty and Elite H-D Light Body on Balb/c 3T3 proliferation evaluated at the 12th and at the 24th hour in culture.

The proliferation is unaffected by the presence of Elite H-D Putty at the 12th and at the 24th hour of culture. The Elite H-D Light Body induces slight inhibition only at the 24th hour, 14% ± 6%. The Express Putty does not induce a significant inhibitory effect. On the contrary, the Express Light Body induces clear inhibition, 62% ± 10% only at the 24th hour (Figure 1B).

In the presence of Permadyne Penta L, after 12 hours of culture, the proliferation of Balb/c 3T3 is lower, 21% ± 9%, in comparison to that found in the control cells. The inhibition is more pronounced, 70% ± 7% after 24 hours of culture. In the presence of Impregum Penta, the inhibition is 37% ± 4% at the 12th hour, and 58% ± 5% at the 24th hour (Figure 1C).

### 2.2. Direct effect of vinylpolysiloxane and polyether on human gingival fibroblasts proliferation

**Figure 2:**
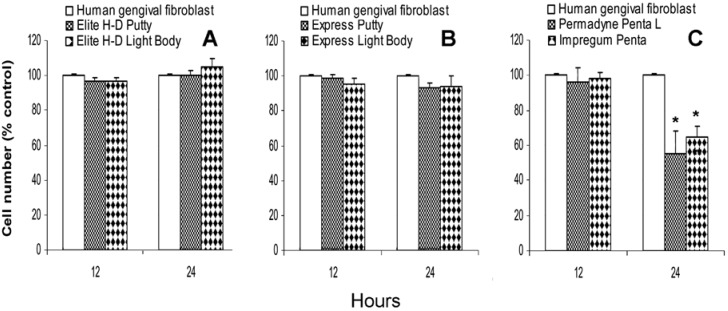
Direct effect of Elite H-D Putty, Elite H-D Light Body (A), Express Putty, Express Light Body (B), and Permadyne Penta L and Impregum Penta (C) on the *in vitro* proliferation of human gingival fibroblasts. Data are expressed as a percentage of the untreated cells. Each graph averages the results from at least four measurements. The data are expressed as a percentage of the untreated cells. Each graph averages the results from at least four measurements. *P <0.05. **P <0.01. One way ANOVA test and Bonferroni multiple comparison test.

The Elite H-D Putty, Express Putty, Elite H-D Light Body and Express Light Body do not inhibit cell proliferation of human gingival fibroblasts at the 12th and 24th hour (Figure 2A and Figure 2B). Permadyne Penta L and Impregum Penta do not inhibit the proliferation of human gingival fibroblasts at the12th hour of culture. At the 24th hour, Permadyne Penta L produces clear inhibition, 45% ± 7.5% and Impregum Penta 35% ± 5% (Figure 2C).

### 2.3. Indirect effect of impression material on Balb/c 3T3 viability

**Figure 3:**
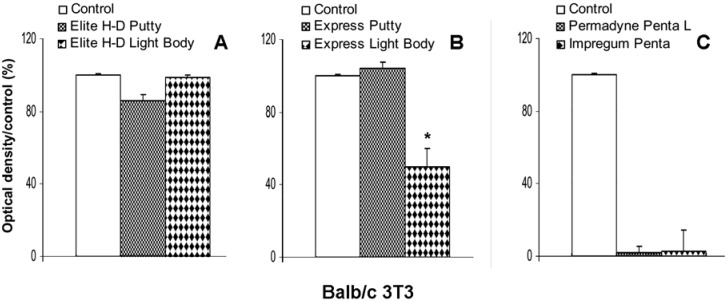
Indirect effect of eluates of vinylpolysiloxane - Elite H-D Putty, Elite H-D Light Body, Figure (A), Express Putty and Express Light Body (B) - Permadyne Penta L and Impregum Penta (C) obtained after 24h of incubation on viability (evaluated by MTT tests) of Balb/c 3T3 cells. The data are expressed as a percentage of optical density (±SE) compared with the untreated cells. Each graph averages the results from at least four measurements. *P <0.05. One way ANOVA test and Bonferroni multiple comparison test.

The eluates obtained after the incubation for 24 hours of Elite H-D Putty, induce a slight inhibition of cellular viability of Balb/c 3T3, 14% ± 4% (Figure 3A), The Elite H-D Light Body and Express Putty extracts do not cause a decrease of Balb/c 3T3 viability. On the contrary, the Express Light Body produces 47% ± 10% inhibition, in comparison with control cultures (Figure 3B). The extracts of Permadyne Penta and Impregum Penta L produce a complete cytotoxic effect (Figure 3C) on Balb/c 3T3 culture.

### 2.4. Indirect effect of impression material on human gingival fibroblast viability

**Figure 4:**
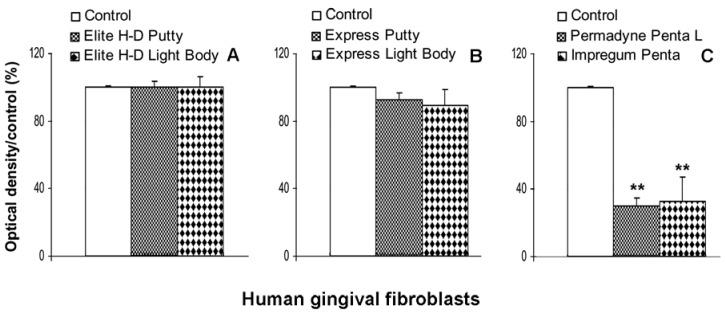
Indirect effect of eluates of vinylpolysiloxane - Elite H-D Putty, Elite H-D Light Body, Figure (A), Express Putty and Express Light Body (B) - Permadyne Penta L and Impregum Penta (C) obtained after 24h of incubation on viability (evaluated by MTT tests) of human gingival fibroblasts. Data are expressed as a percentage of optical density (±SE) compared with the untreated cells. Each graph averages the results from at least four measurements. **P <0.01. One way ANOVA test and Bonferroni multiple comparison test.

No cytotoxic effect was detected with gingival fibroblasts incubated with the eluates of Express Putty, Elite H-D Light Body, Express Putty. The eluates of Express Light Body (Figure 4A and Figure 4B) induces only slight inhibition of cellular viability (Figure 4B). The eluates of Permadyne Penta and Impregum Penta L produce less toxic effect in the gingival fibroblast culture (70% ± 10% and 67% ± 8%), in comparison with Balb/c 3T3 (Figure 4C).

*In vitro* cytotoxicity tests were developed to simulate and predict biological reactions to materials when placed into or on tissue in the body. So particular care should be taken to choose cells and experimental conditions which are relevant for the correlation between *in vivo* and *in vitro* [2]. The objective of the present study was to determine the cytotoxicity of two preparations of dental impression materials - vinylpolysiloxanes and polyethers – and to compare the cytotoxic response of the above materials on the proliferation of two different cell lines cultured *in vitro*. We used Balb/c 3T3 and human gingival fibroblasts. Balb/c 3T3 show good reproducibility and is readily available.

The human gingival fibroblasts, interacting with the dental materials *in vivo*, can present good *in vivo* simulation. Elite H-D Putty (Zhermack) and Express Putty (3M ESPE) do not produce any toxic effect on Balb/c 3T3 and on human gingival fibroblasts. Only Elite H-D Light Body (3M ESPE) induces slight inhibition on Balb/c 3T3 proliferation at the first day of culture. No cytotoxic effect is observed on gingival human fibroblasts. Express Light Body (3M ESPE) is more toxic than Elite H-D Light Body (Zhermack). At the 24th hour of culture, the Balb/c 3T3 proliferation is clearly inhibited. The proliferation of human gingival fibroblasts is unaffected by the presence of Express Light Body (Zhermack). Permadyne Penta L (3M ESPE) and Impregum Penta (3M ESPE) were found to be equally effective in reducing *in vitro* cell proliferation. The Balb/c 3T3 were more sensitive to toxic elements than human gingival fibroblasts. These results confirm previous observations obtained with different preparation of vinylpolysiloxanes and poliethers incubated for short e long times on primary and/or continuous cell line [15,16,17].

## 3. Experimental Section

### 3.1. Impression materials

The dental impression materials were prepared by Zhermack, Badia Polesine, Rovigo, Italy and by 3M ESPE AG Seefeld, Germany. They were supplied in 2 sterile paste forms, base and catalyst, storage at room temperature and dispensed through an auto-mixing cartridge just before used. The preparation of the samples was according to the industrial instructions. The following impression materials were used in this study:
Vinylpolysiloxanes:Elite H-D Putty (Zhermack, Badia Polesine, Rovigo, Italy)Elite H-D Light Body (Zhermack, Badia Polesine, Rovigo, Italy)Express Putty (3M ESPE AG Seefeld, Germany)Express Light Body (3M ESPE AG Seefeld, Germany)Polyethers:Impregum Penta (3M ESPE AG Seefeld, Germany)Permadyne Penta L (3M ESPE AG Seefeld, Germany)


### 3.2. Cell culture

Balb/c 3T3, (embryonic mouse fibroblasts), is a standard cell line widely used for testing early cytotoxicity events. Cells were grown in Dulbecco’s Modified of Eagle’s Medium (DMEM) (Gibco, Grand Island, NY, U.S.A.), containing 4,500 mg/L glucose, 10% fetal calf serum (FCS) (Gibco), 2 mM L-glutamine (Gibco), 50 UI/mL penicillin (Gibco), 50 μg/mL streptomycin (Gibco), and 1 mM Na pyruvate (Gibco), at 37 °C in humidified atmosphere, 95% air and 5% CO_2_.

Human gingival tissues were obtained from adult healthy subject, after providing informed consent, who were undergoing gingivectomy of the molar region. Immediately after removal, the biopsies were placed in “Collection Medium” composed by Hank’s Balanced Salt Solution (HBSS) (Gibco), containing 250 U/mL penicillin, 0.25 mg/mL streptomycin, 0.05 mg/mL gentamycin (Gibco), and 0.0025 mg/mL amphotheracin B (Gibco).The epithelial layer was detached mechanically. The biopsies were finely minced in small pieces and plated in tissue culture flasks (25 cm^2^) (Falcon, BD Bioscience, Milano, Italy) with a thin layer of DMEM, supplemented with 50% FCS (Gibco), 2mM L-glutamine, 1mM Na pyruvate and antibiotics (see “Collection Medium”) at 37 °C in humidified atmosphere, 95% air and 5% CO_2_. The culture medium was made up gradually over the next 3-7 days to 7 mL per 25 cm^2^. Fibroblasts started moving from the explants within two weeks. They were trypsinized (0.25% trypsin); (Gibco) and were subcultured after they had covered at least 50% of the flask surface [28,29]. The cells were routinely cultured in DMEM supplemented with 10% FCS, 50 UI/mL penicillin, 50 μg/mL streptomycin, 2mM L-glutamine and 1mM Na pyruvate at 37 °C in humidified atmosphere, 95% air and 5% CO_2_. In the study, the cultures were used down to the fourth passage.

### 3.3. Cytotoxicity measurements

The cytotoxicity was examined on two different cell lines: Balb/c 3T3 and human gingival fibroblasts cultured *in vitro*. The cells were exposed directly to polymerized impression materials (direct test) or to “eluates” extracted from impression materials (indirect test). The direct and indirect tests of cytotoxicity were carried out according to the methods described in the literature with some modifications [12,13,15,17] and following ISO Document [4,5]. Cell proliferation and cell viability (MTT assay) were the methods used for measuring the *in vitro* cytotoxicity [30]. Results were statistically analyzed with one-way ANOVA test, Bonferroni multiple comparisons test and linear regression correlation test.

#### 3.3.1. Direct test

One centimeter square of the polymerized impression material were placed in the centre of the 60 mm Petri dishes (Falcon, BD Bioscience, Milano, Italy), under sterile conditions. 50 × 10^4^ Balb/c 3T3 or 20 × 10^4^ human gingival fibroblasts were plated in 60 mm Petri dishes (Falcon, BD Bioscience) in a total volume of 5 ml of culture medium with 10% FCS and antibiotics either in the presence or in the absence of impression materials. The dishes without impression materials were used as control. Dishes were incubated at 37 °C under the humidified atmosphere of 95% air and 5% CO_2_.

Balb/c 3T3 cell proliferation was evaluated after 12 and 24 hours of culture according to the method previously described. At each time, the medium was removed, cells in mono-layer were trypsinized (Na-EDTA trypsin) for 7-10 minutes at 37 °C and counted in a haemocytometer (Neubauer). Each count represents the mean of 4 measurements.

Human gingival fibroblasts proliferation was evaluated after 12 and 24 hours. At each time period the medium was removed, cells in mono-layer were trypsinized (Na-EDTA trypsin) (Gibco) for 7-10 minutes at 37 °C and counted in a hemocytometer (Neubauer). Reported values are the mean of 4 replicates.

#### 3.3.2. Indirect test

The indirect test was performed by incubating impression polymerized materials (a 1 cm by 1 cm square) in 60 mm Petri dishes (Falcon, BD Bioscience) in 5 mL of culture medium without serum for 24 hours at 37 °C under sterile conditions. The use of culture medium without serum was adopted in order to avoid the possible interaction or inactivation of substances released by testing materials with serum components [15]. At the end of the incubation, the soluble extracts or eluates of these materials were collected in sterile tubes and enriched with 10% foetal calf serum.

Balb/c 3T3 cells were plated at 50 × 10^3^ per well in 24-well plates (Falcon, BD Bioscience), in 2 mL of culture medium. Human gingival fibroblasts were plated at 20 × 10^3^ per well in 24-well plates (Falcon, BD Bioscience) in 2 mL of culture medium. When the cultures were at the sub-confluence, the culture medium was removed; the cell mono-layer was washed with PBS and then supplemented with 1 mL of extract. Each eluate together with the controls were tested in quadruplicate wells. At the end of the treatment, cellular viability was estimated by MTT assay [30].

#### 3.3.3. MTT test

MTT (3-[4,5-dimethylthiazol-2-yl]-2,5-diphenyltetrazolium bromide) (Sigma, St Louis, MO, U.S.A.) is a water-soluble tetrazolium dye which produces a yellowish solution when dissolved in culture medium or in saline solutions. Only live cells will reduce it to a purple formazan product insoluble in aqueous solutions [30,31]. MTT viability test is based on the amount of formazan generated that is consequently directly proportional to the number of viable cells. The MTT assay is an indirect marker for cytotoxicity.

After 24 hours of cell cultures, in the absence or in the presence of eluates, the medium was removed and 2 mL of growth medium with 100 μL of MTT (5 mg/mL in PBS) were added to the cultures. Subsequently, the cells were incubated at 37 °C for 3 hours in a humidified atmosphere (95% air and 5% CO_2_). At the end of the incubation, 2 mL of dimethyl sulphoxide (DMSO) (Sigma, St Louis, MO, U.S.A.) were added to each well to dissolve purple crystals of formazan. The coloured solution was measured in a spectrophotometer at a wavelength of 540 nm in order to evaluate the optical density, which correlates directly with the number of viable cells.

Reported values are the mean of four measurements and are expressed as percentages of the control values. Results were statistically analyzed with one-way ANOVA test, Bonferroni multiple comparisons test, and linear regression correlation test.

## 4. Conclusions

The present study suggests:

a) *in vitro* cell culture technique can be used to rate the cytotoxic effect of dental impression materials;
b) Balb/c 3T3, a continuous cell line, is more sensitive to cytoxic effects of dental materials than human gingival fibroblasts, a finite cell line;
c) the direct *in vitro* tests are able to evaluate the toxic effects of the materials on adjacent cells. In the indirect test, the effect of “eluates” derived from the incubation of dental materials in culture medium, might be precisely quantified;
c) both direct and indirect tests demonstrate that the vinylpolysiloxanes materials are less toxic than polyethers in our experimental conditions;
d) among the vinylpolysiloxanes materials, Elite H-D Putty and Express Putty do not reduce Balb/c 3T3 and human gingival fibroblasts proliferation. The Express Light Body is found more toxic than Elite H-D Light Body;
e) continuous cell line such Blab/c 3T3 are recommended as cells of choice for preliminary screening dental materials *in vitro*
f) the biotoxicity studies ideally should employ human diploid gingival fibroblasts because the cells retain specialized features.

For *in vitro* cytotoxicity screening, the recommended testing methods include: direct or indirect test. The test must be of a short duration, simple and low expensive. In dental treatment it is advantageous to maintain maximum tissue vitality and cytotoxic reactions must be prevented, hence the requirement to screen all dental materials before they are used clinically.
